# Prediction of Pleural Invasion in Challenging Non-Small-Cell Lung Cancer Patients Using Serum and Imaging Markers

**DOI:** 10.1155/2020/6430459

**Published:** 2020-02-07

**Authors:** Kaibin Zhu, Lantao Chen, Changjun He, Yaoguo Lang, Xianglong Kong, Changfa Qu, Shidong Xu

**Affiliations:** Department of Thoracic Surgery, Harbin Medical University Cancer Hospital, Harbin 150001, China

## Abstract

*Introduction*. Preoperative detection of pleural invasion in lung cancer patients is key to curative surgical treatment. We tried to predict pleural invasion in non-small-cell lung cancer patients with <100 ml pleural fluid.

**Methods:**

Patients admitted from August 1, 2011, to December 31, 2018, were retrospectively retrieved. Records of serum and imaging markers were analyzed.

**Results:**

Among 7004 patients who received surgery, 43 cases with <100 ml pleural fluid who had pleural invasion were included, and another 108 cases without pleural invasion were enrolled as controls. There were no differences in squamous cell carcinoma antigen (SCC) or neuron-specific enolase (NSE) values between the pleural invasion and noninvasion groups (*p* = 0.30 and 0.14, respectively), but there were significant differences in carcinoembryonic antigen (CEA) and cytokeratin 19 fragment (CYFRA21-1) values (*p* = 0.30 and 0.14, respectively), but there were significant differences in carcinoembryonic antigen (CEA) and cytokeratin 19 fragment (CYFRA21-1) values (*p* = 0.30 and 0.14, respectively), but there were significant differences in carcinoembryonic antigen (CEA) and cytokeratin 19 fragment (CYFRA21-1) values (*p* = 0.30 and 0.14, respectively), but there were significant differences in carcinoembryonic antigen (CEA) and cytokeratin 19 fragment (CYFRA21-1) values (*p* = 0.30 and 0.14, respectively), but there were significant differences in carcinoembryonic antigen (CEA) and cytokeratin 19 fragment (CYFRA21-1) values (*p* = 0.30 and 0.14, respectively), but there were significant differences in carcinoembryonic antigen (CEA) and cytokeratin 19 fragment (CYFRA21-1) values (*p* = 0.30 and 0.14, respectively), but there were significant differences in carcinoembryonic antigen (CEA) and cytokeratin 19 fragment (CYFRA21-1) values (

**Conclusions:**

Serum CEA and CYFRA21-1, location of original lung cancer (right mid lobe), maximum diameter, CT-detectable pleural fluid, pleural sign by CT, and PET/CT-predicted pleural invasion were good markers for the prediction of pleural invasion in non-small-cell lung cancer patients.

## 1. Introduction

Lung cancer has the highest incidence among all cancers worldwide, and over 1.8 million new cases are diagnosed each year [1]. The overall survival for clinical stage IIIB non-small-cell lung cancer in 5 years remains at 26% [2]. Correct clinical staging, although challenging, defines the extent of the disease, determines treatment, and decides survival. Thus, it is essential to distinguish between operable and nonoperable patients.

In the group of non-small-cell lung cancer patients, pleural invasion is considered a poor-prognostic factor, and those patients are not recommended for curative surgical treatments alone [3, 4]. However, despite the improvement in imaging techniques, proximal tumor extension may be underestimated before surgery. Imaging techniques such as computed tomography (CT) [5], positron emission tomography/computed tomography (PET/CT), magnetic resonance imaging (MRI) [6, 7], and ultrasound have been employed in the diagnosis and prognosis of lung cancer. However, the diagnosis of proximal pleural invasion, especially in those with nondetectable or small volume of pleural fluid by CT measurement, which is beyond the minimum detectable limit in quite many cases, is still challenging based on imaging markers.

Squamous cell carcinoma antigen (SCC), neuron-specific enolase (NSE), carcinoembryonic antigen (CEA), and cytokeratin 19 fragment (CYFRA21-1) have been reported to be valuable in the diagnosis and prognosis of lung cancer [8, 9]. However, no study investigated their values in the prediction of pleural invasion in non-small-cell lung cancer patients. Due to the lack of reliable tools to predict pleural invasion, originally scheduled curative surgical procedures may ultimately be restricted to an exploratory thoracotomy (ET) because of pleural invasion confirmed only during surgery [10].

In order to avoid unnecessary ET while increasing the rate of curative surgical procedures, we sought to predict pleural invasion in non-small-cell lung cancer patients without distant metastasis and with small volume of pleural fluid before surgery using serum and imaging markers.

## 2. Methods

This retrospective cohort study was conducted at Harbin Medical University Cancer Hospital, a tertiary hospital and regional specialized center for the treatment of cancer in Northeast China. Institutional Ethics Committee approval was obtained before the start of the study. Informed consent was obtained from all enrolled patients.

All clinical records of patients who received surgical treatment for lung cancer between August 1, 2011, and December 30, 2018, in the Department of Thoracic Surgery of Harbin Medical University Cancer Hospital were retrieved and reviewed. Included patients should meet all of the following criteria: (1) received lung surgery for the treatment of non-small-cell lung cancer and had no previous treatment including any surgeries or chemotherapy or radiotherapy; (2) preoperative serum test records available, including SCC, NSE, CEA, and CYFRA21-1; (3) preoperative diagnostic CT records showing the size and location of the lesion, with nondetectable or small volume of pleural fluids (<100 ml by CT measurement) [11, 12]; (4) no distant metastases (pleural invasion was not necessarily considered to be proximal metastasis since the location is next to the lung [13, 14]) confirmed by bronchoscopy, contrast-enhanced CT scan of the chest and brain, bone ECT, color Doppler ultrasonography of the superficial lymph nodes, liver, and adrenal, and PET [15]; and (5) postoperative pathology findings available. The pleural-invaded group included patients with pleural invasion lesions found during surgery and confirmed by pathology, whereas those in the control group were without pleural invasion lesions confirmed during surgery or suspected lesions confirmed to be pleural noninvasive lesions by pathology. Both groups were confirmed for original lung cancer lesions [16].

The surgeries including video-assisted thoracoscopic surgery (VATS) or thoracotomy were performed by the same group of surgeons, with experience of similar surgeries for over 10 years before the start date of this study. The imaging results were interpreted by the same group of physicians with over 10 years of expertise in lung cancer before the start date of this study.

Pleural sign was determined by CT (SOMATOM Definition AS+, Siemens Healthineers, Germany) as previously reported [17]. Briefly, four types of findings were considered positive: one linear pleural tag with soft tissue component at the pleural end, one cord-like pleural tags with soft tissue component at the pleural end, tumor about the pleura, and tumor push pleura.

Fasting peripheral venous blood samples (3.0 ml) were collected from all patients before treatment and kept at 2-8°C. Serum was separated by centrifugation at 4000 rpm for 10 minutes within 2 hours of collection. CEA, CYFRA21-1, and NSE were detected by an automatic electrochemical luminescence analyzer (Cobas e602, Roche, Germany), with a normal upper limit of 1.5 ng/ml for SCC, 15.2 ng/ml for NSE, 5 ng/ml for CEA, and 3.3 ng/ml for CYFRA21-1. All tests were conducted according to instrument operating manuals [8].

### 2.1. Statistical Analysis

Discrete data were expressed as the number of cases (percentages) and analyzed using *χ*^2^ test or Fisher's exact test, along with odds ratio (OR) and 95% confidence interval (95% CI), whichever was applicable. Continuous data were shown as mean ± standard deviation (SD) and were analyzed using *t*-test. Area under the Receiver Operating Characteristic (ROC) curve was used to show the value of prediction. SPSS24.0 (IBM Corp., Armonk, NY) was used for statistical analysis. A two-tailed *p* < 0.05 is considered significantly different.

## 3. Results

During the preset study period, 7004 patients received surgery treatments for lung cancer at our hospital. 214 cases had pleural lesion pathology results available. 32 of them had nonmalignant pleural tissues, and another 135 of them had >100 ml pleural fluid by CT evaluation and were highly suspected to have pleural invasion already. Excluding another 4 cases with incomplete serum marker data, a total of 43 patients qualified for the inclusion criteria, including 19 males and 24 females. 108 cases of pleural noninvasion adenocarcinoma cases (confirmed during surgery) with complete serum and imaging marker data admitted between March 15, 2018, and December 30, 2018, were enrolled as controls. There was no significant difference in age or gender between the two groups (*p* > 0.05, Table 1).

### 3.1. The Relationship between Serum Markers and Pleural Invasion

**Figure 1:**
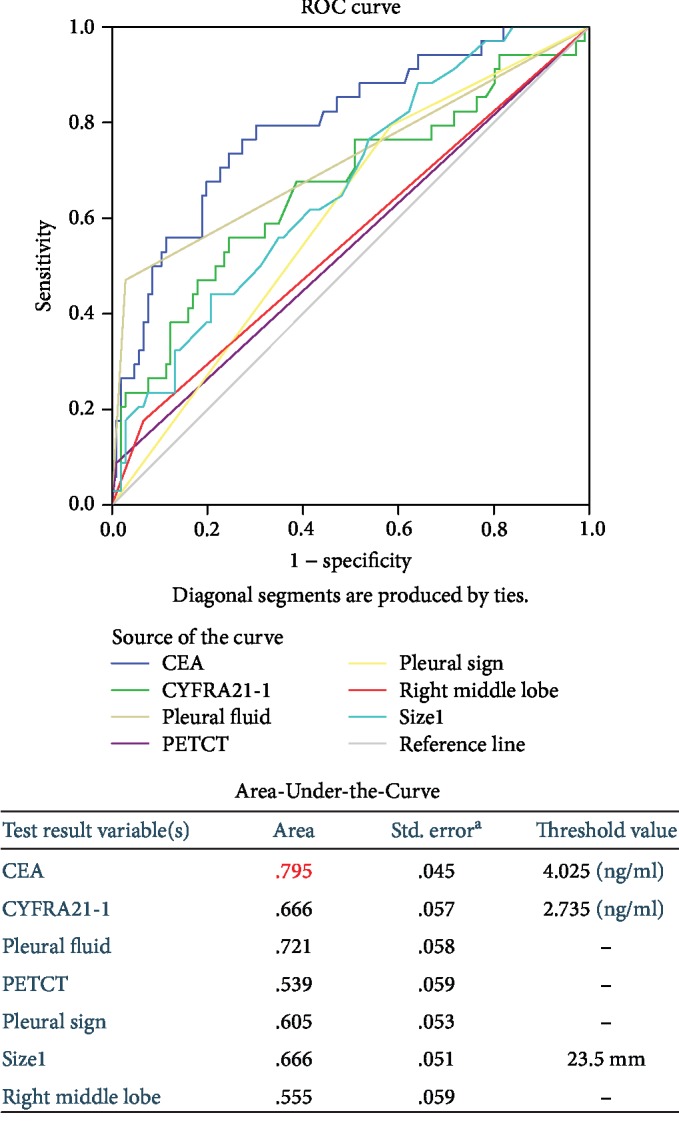
Receiver Operating Characteristic (ROC) curve of individual serum and imaging markers. Upper panel: ROC curves of each marker. Lower panel: areas below the curve and cutoff values of each marker.

There were no differences in SCC or NSE values between the pleural invasion and noninvasion groups (*p* = 0.30 and 0.14, respectively), but there were significant differences in CEA and CYFRA21-1 values (*p* < 0.01 and 0.01, respectively, Table 2). The OR (8.73 (3.88, 19.65) and 3.53 (1.56, 7.98), respectively), sensitivity (74.42% and 51.16%, respectively), specificity (75.00% and 76.85%, respectively), positive predictive value (PPV) (54.24% and 46.81%, respectively), and negative predictive value (NPV) (88.04% and 79.81%, respectively) of each serum marker were shown in Table 2. Using ROC curve analysis, the Area-Under-the-Curve (AUC) was 0.795 for CEA and 0.666 for CYFRA21-1. The predicted optimal cutoff values for pleural invasion were 4.025 ng/ml for CEA and 2.735 ng/ml for CYFRA21-1 (Figure 1).

### 3.2. The Relationship between Imaging Markers and Pleural Invasion

There were significant differences in the location of original lung cancer (right mid lobe, *p* = 0.01), maximum diameter (*p* < 0.01), volume of pleural fluid measured by CT (nondetectable vs. detectable fluid <100 ml, *p* < 0.01), pleural sign (*p* = 0.03), and PET/CT-predicted pleural invasion (*p* = 0.02) between the pleural invasion and noninvasion groups. The OR, sensitivity, specificity, PPV, and NPV of each imaging marker were shown in Table 2. Using ROC curve analysis, the AUC was 0.555 for location, 0.666 for maximum diameter, 0.605 for pleural sign, 0.539 for PET/CT prediction, and 0.721 for pleural fluid. The predicted optimal cutoff value for pleural invasion was 23.5 mm for the max diameter of the original lung cancer lesion (Figure 1).

### 3.3. Combinations of Multiple Markers in the Prediction of Pleural Invasion

Since the majority of single markers has a medium AUC, we went further to test if combinations of more markers could improve the AUC. When any 2 of the 7 markers with *p* < 0.05 were combined, the maximum AUC was achieved with the combination of CEA and pleural fluid (0.811, Supplement [Supplementary-material supplementary-material-1]). When any 3 of the 7 markers with *p* < 0.05 were combined, the maximum AUC was achieved with the combination of CEA, pleural fluid, and location of the lung lesion (0.832, Supplement [Supplementary-material supplementary-material-1]). When any 4 of the 7 markers with *p* < 0.05 were combined, the maximum AUC was achieved with the combination of CEA, pleural fluid, pleural sign, and location of the lung lesion (0.853, Supplement [Supplementary-material supplementary-material-1]). When any 5 of the 7 markers with *p* < 0.05 were combined, the maximum AUC was achieved with the combination of CEA, pleural fluid, PET/CT prediction, pleural sign, and location of the lung lesion (0.859, Supplement [Supplementary-material supplementary-material-1]). When any 6 of the 7 markers with *p* < 0.05 were combined, the maximum AUC was achieved with the combination of CEA, CYFRA21-1, pleural fluid, PET/CT prediction, pleural sign, and location of the lung lesion (0.865, Supplement [Supplementary-material supplementary-material-1]). When all 7 markers were combined, the AUC was 0.860 (Supplement [Supplementary-material supplementary-material-1]).

Furthermore, we found that pleural fluid and pleural sign can be included in a logistic regression equation for prediction of pleural invasion, i.e., *p* = 1/[1 + *e* − (−4.208 + 3.125PleuralFluid + 1.610PleuralSign)], where PleuralFluid or PleuralSign equals 0 if absent and equals 1 if present.

## 4. Discussion

Surgery remains the first choice in the treatment of non-small-cell lung cancer in early stages. However, there is still no effective test to detect small lesion of pleural invasion before surgery. The rate of ET has been reported to be 4.0%~12.25% [10], which, ideally, was suggested to be 1% or less [10]. On the other hand, a zero ET rate based entirely on unreliable preoperative imaging markers or noninvasive procedures might eliminate the surgical opportunity that patients with actually resectable tumors may have.

According to the latest TNM staging system, pleural invasion of NSCLC has been adopted as a T impact factor in the TMN staging system of UICC since the 1970s [18], although in some reports pleural invasion was classified as M1a and accounted for 7%-15% of lung cancer cases [19], with a 5-year survival rate of 16% [20].

This is the first study that comprehensively investigated the role of serum and imaging markers in the prediction of pleural invasion in non-small-cell lung cancer patients with small volume (<100 ml) to nondetectable pleural fluid by CT evaluation. As was shown in our study, a pleural invasion is often seen in adenocarcinomas because of their peripheral location [21] and accompanied by pleural sign [17]. The latter phenomenon is indicative of the pleural invasion process of the original lung lesion. Pleural-invaded cases at early stage may only present with thickened pleura under CT scan. PET/CT may further show a “hot” status within the thickened pleura, which is more helpful with the diagnosis of pleural invasion. In a prospective clinical trial, it was shown that preoperative PET/CT for staging of NSCLC reduced both total thoracotomies and futile thoracotomies [22]. In another retrospective clinical study, high-resolution computed tomography (HRCT) findings and fluorine-18-fluorodeoxyglucose ((18) F-FDG) uptake have been employed for risk stratification of visceral pleural invasion by lung adenocarcinoma [23].

The size of the lung lesion has been related with the prognosis of NSCLC patients using a pathological tumor size of 30 mm as a cutoff [24]. In our present study, both size and location of the lesion detected by CT were related with the occurrence of pleural invasion, which, besides the reported pathology findings after surgery, showed the importance of tumor size and location in the development of pleural invasion.

The presence of malignant pleural effusion (MPE) is an adverse complication that shortens the life expectancy [25]. A large volume of pleural fluid is often seen in pleural-invaded cases at late stage but seldom in pleural-invaded cases at early stage or in noninvaded cases. On the other hand, some surgeons believe even small volumes of pleural fluid are indicative of pleural invasion, and thus, those patients are ineligible for curative surgical procedures, although some cases in fact have no pleural invasion. A small volume of pleural fluid is not easy to be collected for analysis even under ultrasound guide. It may also be caused by hypoproteinemia or inflammation and therefore should not be a contraindication for surgical treatment. However, in challenging cases, i.e., in the absence of MPE, or only a small volume of pleural fluids, it is often difficult to diagnose pleural invasion based only on imaging markers, considering the low sensitivity and specificity of individual markers (Table 2). Therefore, we analyzed both serum markers and imaging markers, as well as combinations of different markers in a pleural-invaded cohort and a noninvaded cohort, trying to figure out a better and comprehensive predictive method.

The markers analyzed in this study were all routine tests before surgery and add no more costs to the treatment of patients. Unexpectedly, although there were significant differences in 2 serum markers and 5 imaging markers between the invaded and noninvaded cohorts, we found that serum CEA had the largest AUC. Due to the low sensitivity and specificity of single markers for lung cancer, a combination of different markers could increase the accuracy of diagnosis and staging [26].

In leptomeningeal metastasis cases (LM), cerebrospinal fluid CEA and CYFRA21-1 levels have high accuracy for differentiating LM from nonmalignant neurological diseases, whereas serum CYFRA21-1 is of poor diagnostic value [27]. In another study, increase in a combination of cancer markers in pleural fluid is highly suggestive of pleural invasion, and cancer markers in serum samples of pleural-invaded cases were found to increase, too [26]. Both studies reflected the potential value of CEA and CYFRA21-1 in the diagnosis of malignant metastasis and the probable different values between serum and other body fluids markers.

## 5. Conclusion

In summary, we found that serum CEA and CYFRA21-1 and the location of original lung cancer (right mid lobe), maximum diameter, CT-detectable pleural fluid, pleural sign by CT, and PET/CT-predicted pleural invasion were good markers for the prediction of pleural invasion in non-small-cell lung cancer patients. When considering in a comprehensive manner, the maximum predictive value was achieved with the combination of CEA, CYFRA21-1, detectable pleural fluid, PET/CT-predicted pleural invasion, pleural sign, and lung lesion at the right mid lobe. Furthermore, detection of pleural fluid and pleural sign can be included in a logistic regression equation for prediction of pleural invasion.

## Figures and Tables

**Table 1:** Clinical characteristics of enrolled patients.

Cases	Pleural invasion	Nonpleural invasion	OR (95% CI)^#^	*p* value
Male	19	38	0.73 (0.35, 1.54)	0.30^∗^
Age (years)	56.4 ± 7.4	58.6 ± 7.5	—	0.10^∗∗^
Total	43	108	—	—

^∗^ *χ* ^2^ test, number of patients. ^∗∗^t-test, mean ± SD. ^#^Odds ratio (95% confidence interval).

**Table 2:** Relationship between serum or imaging markers and pleural invasion.

Markers	Pleural invasion	Nonpleural invasion	OR (95% CI)	*p* value	Sensitivity (%)	Specificity (%)	PPV (%)	NPV (%)
SCC value (ng/ml, mean ± SD)	1.65 ± 3.52	0.93 ± 0.88	—	0.05^$^	—	—	—	—
SCC abnormal	5 (38)	7 (101)	1.90 (0.57, 6.35)	0.30^#^	11.63	93.52	41.67	72.66
NSE value (ng/ml, mean ± SD)	16.8 ± 14.8	14.3 ± 3.4	—	0.11^$^	—	—	—	—
NSE abnormal	19 (24)	34 (74)	1.72 (0.83, 3.56)	0.14^#^	44.19	68.52	35.85	75.51
CEA value (ng/ml, mean ± SD)	35.0 ± 62.8	7.35 ± 15.10	—	**<0.01** ^$^	—	—	—	—
CEA abnormal	32 (11)	27 (81)	8.73 (3.88, 19.65)	**<0.01** ^#^	74.42	75.00	54.24	88.04
CYFRA21-1 value (ng/ml, mean ± SD)	5.34 ± 7.55	2.87 ± 1.87	—	**<0.01** ^$^	—	—	—	—
CYFRA21-1 abnormal	22 (21)	25 (83)	3.53 (1.56, 7.98)	**<0.01** ^#^	51.16	76.85	46.81	79.81
Right mid lobe	9 (34)	7 (101)	3.82 (1.32, 11.04)	**0.01** ^#^	20.93	93.52	56.25	74.81
Max diameter (mm, mean ± SD)	34.7 ± 19.9	24.8 ± 12.3	—	**<0.01** ^$^	—	—	—	—
Max diameter^∗^	27 (16)	47 (61)	2.19 (1.06, 4.53)	**0.03** ^#^	62.79	56.48	36.49	79.22
Pleural fluid	19 (24)	3 (105)	27.71 (7.58, 101.25)	**<0.01** ^#^	55.81	2.78	18.60	13.64
Pleural sign	33 (10)	62 (46)	2.45 (1.10, 5.47)	**0.03** ^#^	76.74	42.59	34.74	82.14
PET predicted	5 (38)	1 (107)	14.08 (1.59, 124.39)	**0.02** ^#^	11.63	99.07	83.33	73.79
Total	43	108	—	—	—	—	—	—

Integer numbers in brackets represent cases with normal value. SCC: squamous cell carcinoma antigen; NSE: neuron-specific enolase; CEA: carcinoembryonic antigen; CYFRA21-1: cytokeratin 19 fragment; OR: odds ratio; PPV: positive predictive value; NPV: negative predictive value. ^$^*t*-test. ^#^*χ*^2^ test or Fisher's exact test. ^∗^Max diameter at 23.5 mm cutoff for positivity.

## Data Availability

Original data could be obtained by contacting the corresponding author.

## Supplementary Materials

Supplementary Materials Supplement Figure 1: Receiver Operating Characteristic (ROC) curve of combination of any two serum and imaging markers. Supplement Figure 2: Receiver Operating Characteristic (ROC) curve of combination of any three serum and imaging markers. Supplement Figure 3: Receiver Operating Characteristic (ROC) curve of combination of any 4 serum and imaging markers. Supplement Figure 4: Receiver Operating Characteristic (ROC) curve of combination of any 5 serum and imaging markers. Supplement Figure 5: Receiver Operating Characteristic (ROC) curve of combination of any 6 or all 7 serum and imaging markers.
