# Supplementary material for: Prediction of Pleural Invasion in Challenging Non-Small-Cell Lung Cancer Patients Using Serum and Imaging Markers
Source: Dis Markers. 2020 Feb 7;2020:6430459. doi: 10.1155/2020/6430459 (PMC7029264; doi:10.1155/2020/6430459)
Supplement: Supplementary Materials — Supplement Figure 1: Receiver Operating Characteristic (ROC) curve of combination of any two serum and imaging markers. Supplement Figure 2: Receiver Operating Characteristic (ROC) curve of combination of any three serum and imaging markers. Supplement Figure 3: Receiver Operating Characteristic (ROC) curve of combination of any 4 serum and imaging markers. Supplement Figure 4: Receiver Operating Characteristic (ROC) curve of combination of any 5 serum and imaging markers. Supplement Figure 5: Receiver Operating Characteristic (ROC) curve of combination of any 6 or all 7 serum and imaging markers. [file 6430459.f1.ppt]

## Slide 1
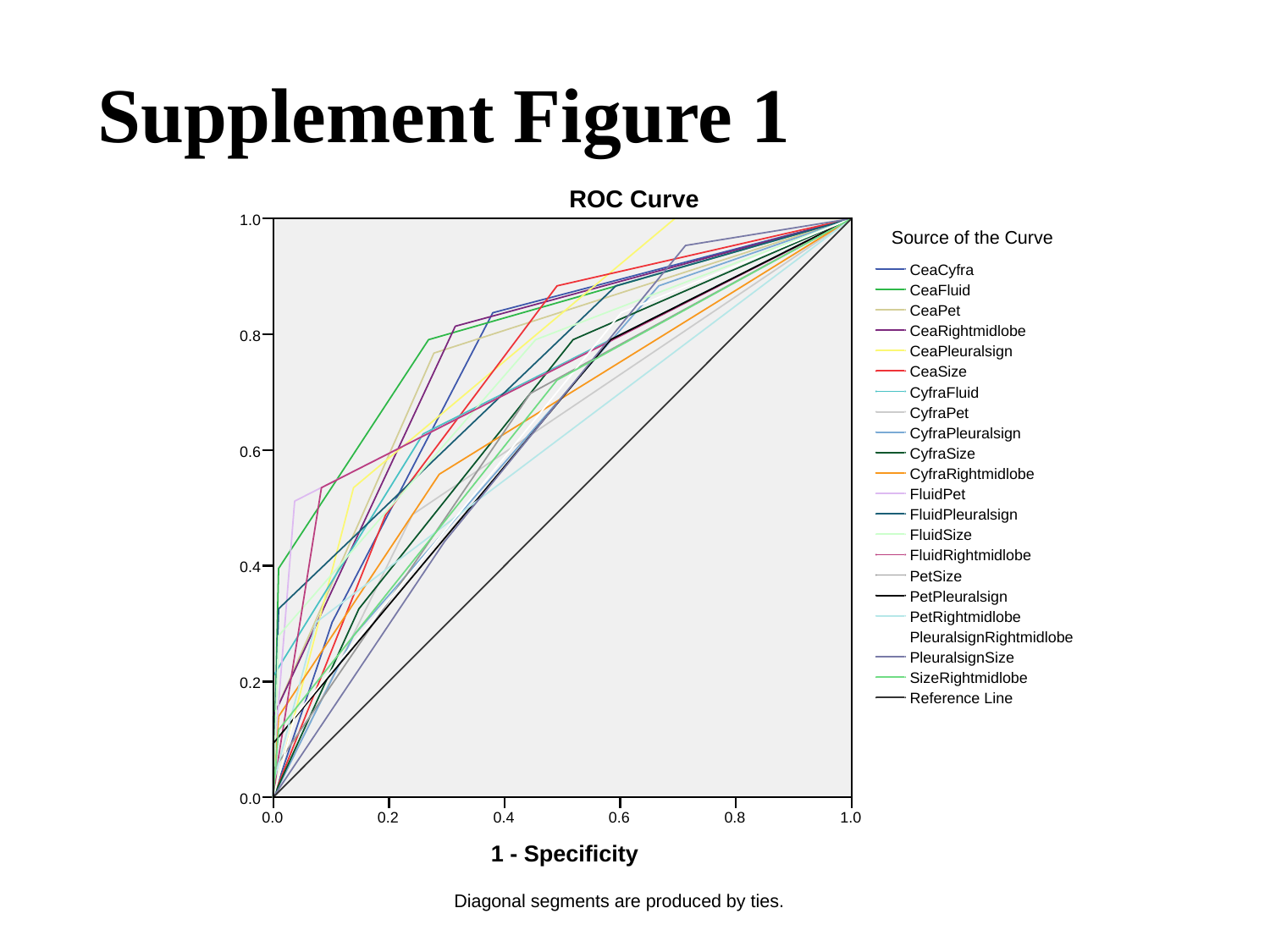

# Supplement Figure 1

## Slide 2
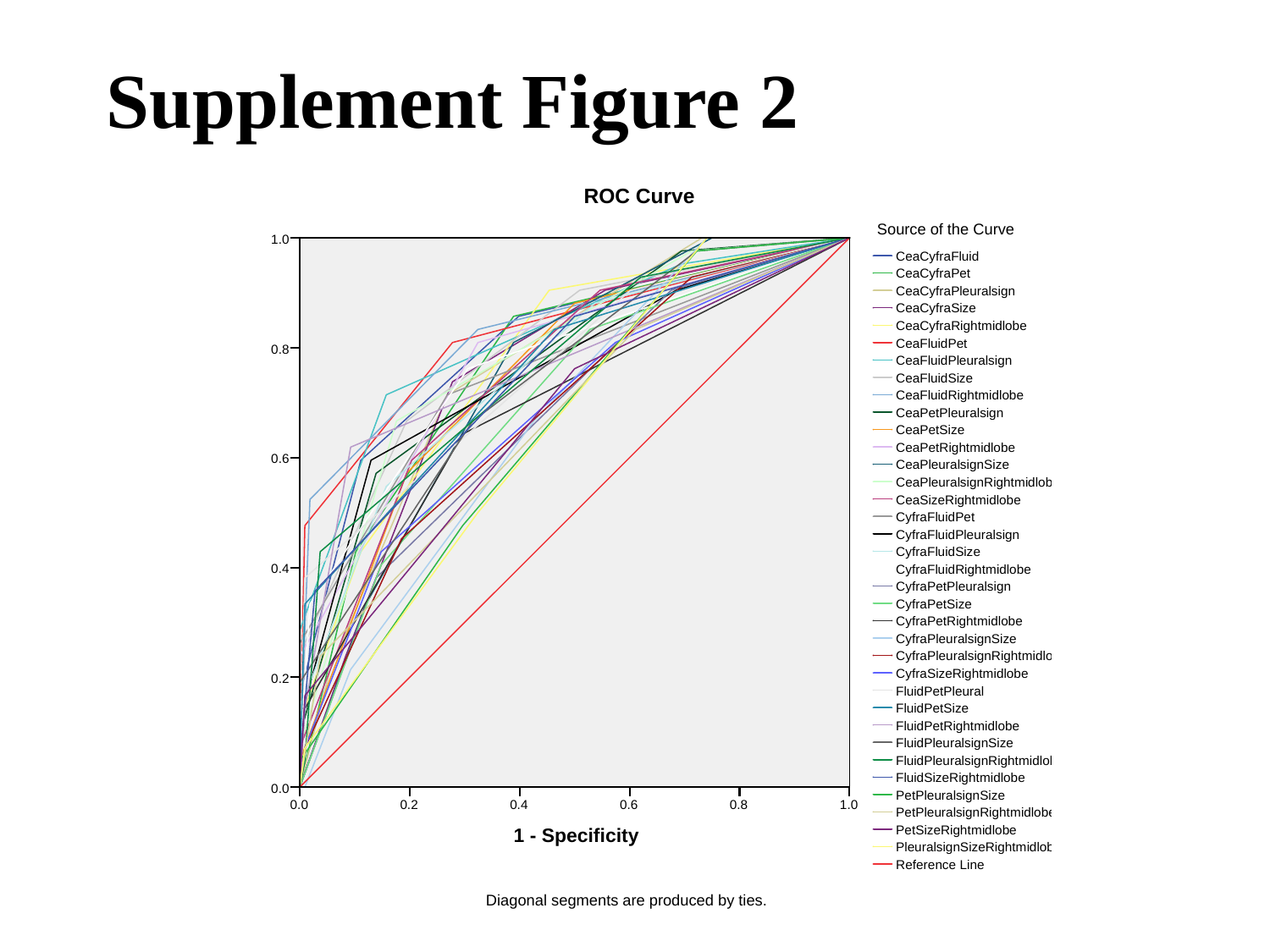

# Supplement Figure 2

## Slide 3
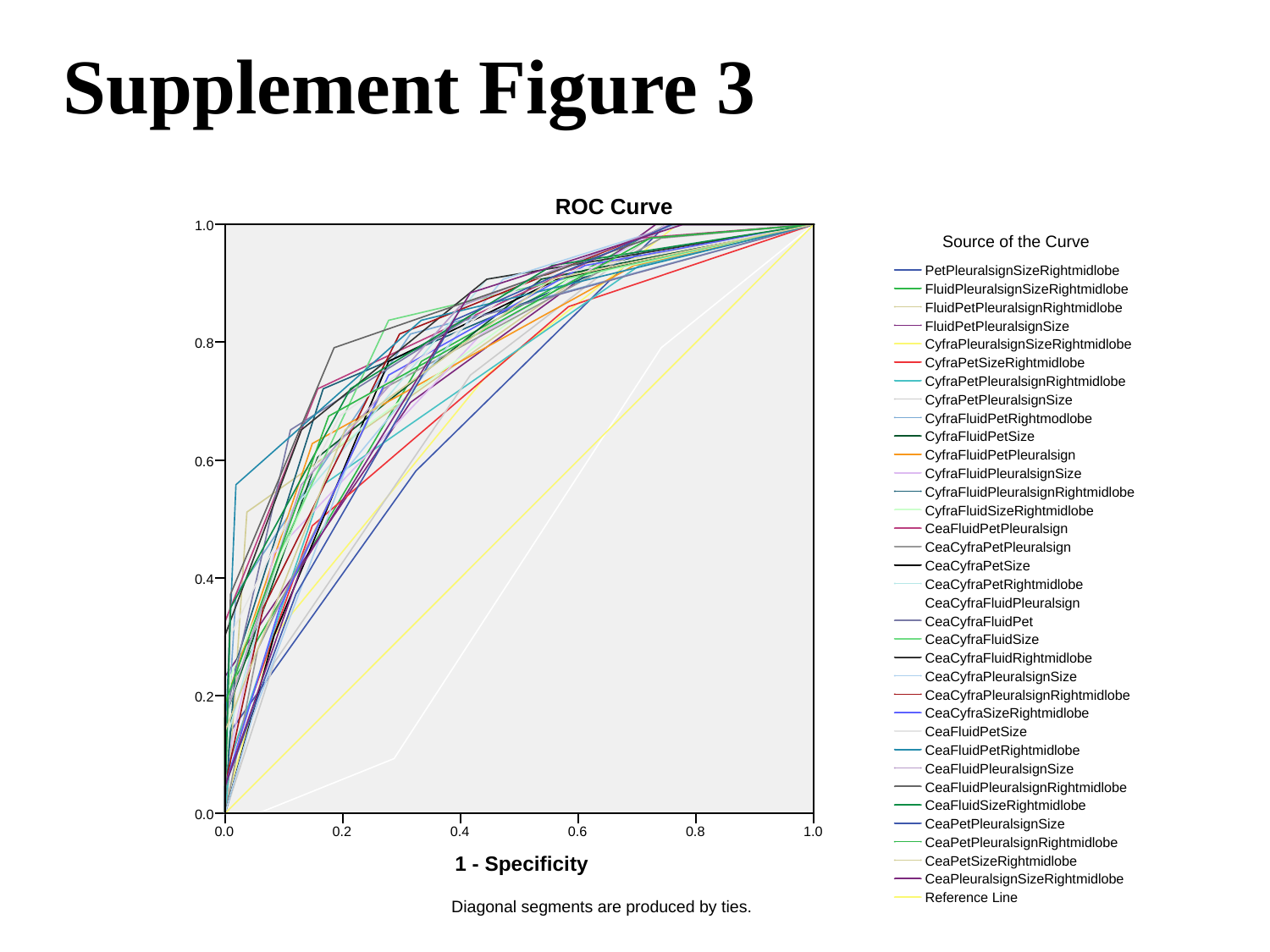

# Supplement Figure 3

## Slide 4
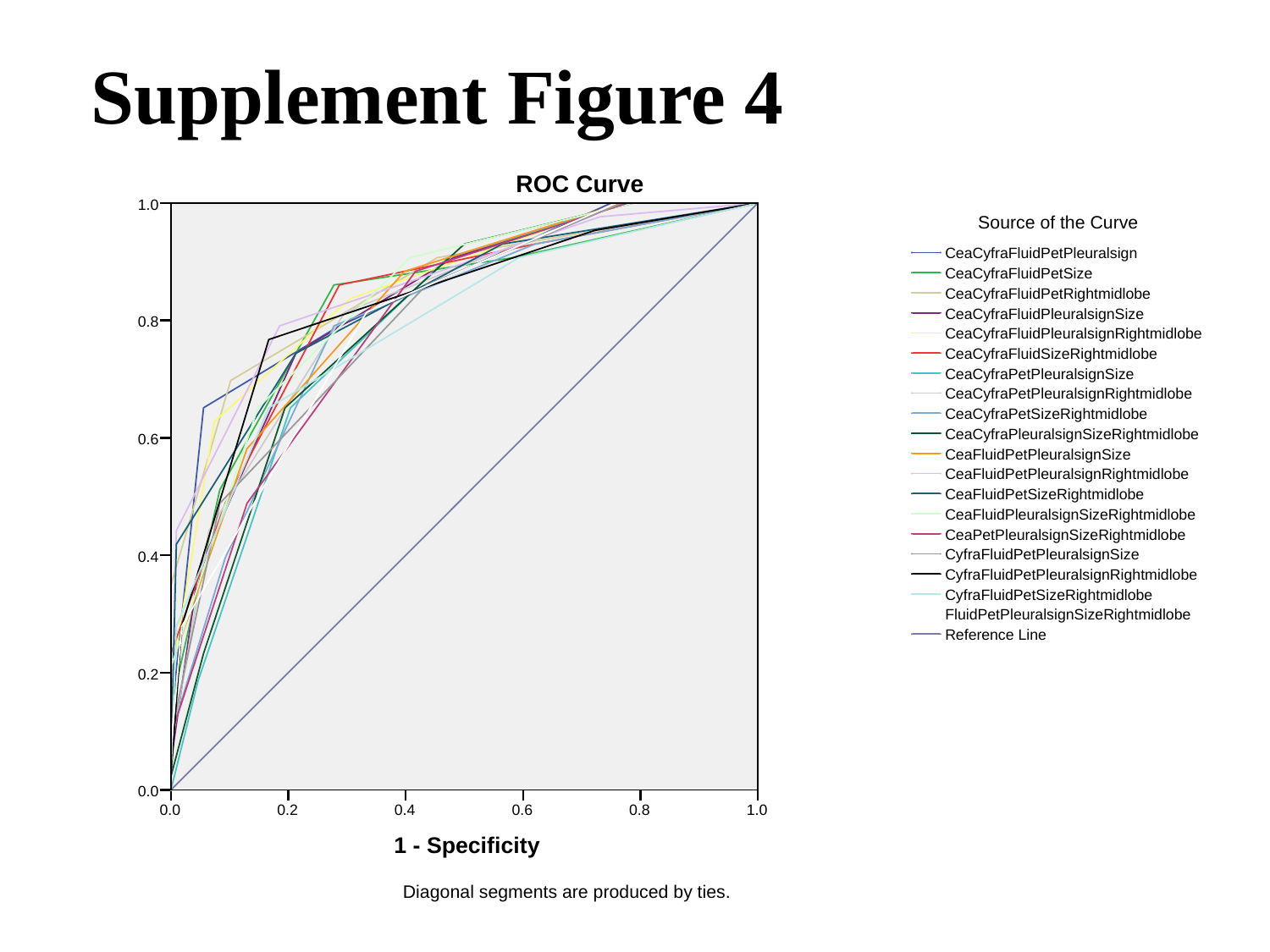

# Supplement Figure 4

## Slide 5
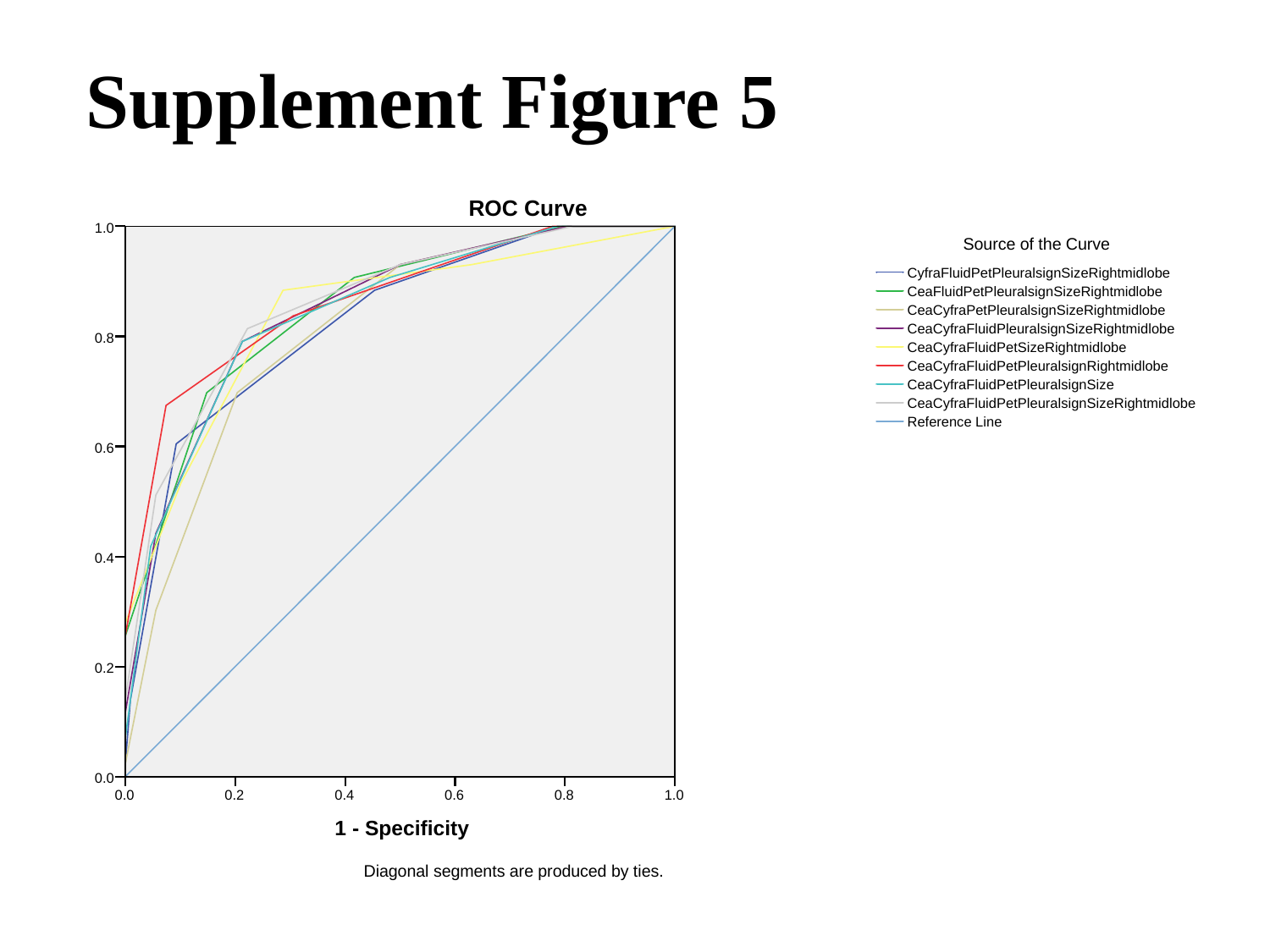

# Supplement Figure 5
